# Effectiveness of case management and psychological intervention nursing model in bariatric surgery patients

**DOI:** 10.3389/fsurg.2025.1614595

**Published:** 2025-06-24

**Authors:** Qin Sun, Aimei Jia, Yuan Zhang, Dianyuan Liang, Ke Song, Norshafarina Shari

**Affiliations:** ^1^ School of Management/Key Laboratory of Digital-Intelligent Disease Surveillance and Health Governance, North Sichuan Medical College, Nanchong, China; ^2^ School of Graduate Studies, Postgraduate Center, Management and Science University, Shah Alam, Malaysia; ^3^ Department of Enterogastric Surgery, Affiliated Hospital of North Sichuan Medical College, Nanchong, China

**Keywords:** case management, bariatric surgery, obesity, psychological intervention, postoperative outcomes

## Abstract

**Objectives:**

This study aimed to analyze the significance and value of the case management and psychological intervention model in bariatric surgery patients.

**Methods:**

A retrospective study was conducted on 100 patients who underwent bariatric surgery admitted to the Affiliated Hospital of North Sichuan Medical College from January 1, 2021, to December 31, 2023. The patients were divided into two groups based on the nursing model. The control group (*n* = 50) received conventional nursing, while the experimental group (*n* = 50) was treated with case management combined with psychological intervention nursing. The changes in physical indicators, patient satisfaction, psychological condition, and quality of life were compared between the two groups.

**Results:**

The body mass index (BMI) of the experimental group at 12 months post-surgery was significantly different from those of the control group (*P* < 0.05). Regarding psychological assessment, the anxiety and depression scores of patients showed significant differences at the initial outpatient visit and discharge day (*P* < 0.05). Quality of life indicators (physical function, bodily pain, emotional function, social function) were statistically significantly different at 6 months post-surgery (*P* < 0.05).

**Conclusion:**

The case management and psychological intervention model can significantly promote weight reduction, psychological assessments, and quality of life functions in patients after bariatric surgery.

## Introduction

As society progresses and living standards improve, changes in dietary habits and lifestyle have contributed to the rising number of obese individuals (1). Obesity is a major risk factor for numerous diseases and significantly impacts individuals' quality of life (2). The effectiveness of bariatric surgery in treating obesity and related metabolic disorders has been fully confirmed. It is currently the best surgical approach for reducing body mass index (BMI) and can decrease the overall mortality rate in obese individuals (3). Obese individuals often experience anxiety, depression, and other psychological issues, which may influence the outcomes of bariatric surgery (4). Current studies demonstrate that active psychological interventions can alleviate negative emotions in obese patients, improve the psychological state of post- surgery patients, enhance adherence to medical advice and physical activity, and improve patients' quality of life (5). Case management is an innovative healthcare management model involving the collaborative participation of case managers, healthcare professionals, and patients. Led by the case manager, this multidisciplinary and multi-professional approach offers specialized, personalized, continuous, and comprehensive medical services to patients and their families (6). However, there is limited evidence exists regarding the application of case management combined with psychological intervention in patients who underwent bariatric surgery. This approach involves providing different psychological interventions based on each patient's unique psychological characteristics, ensuring specialized, personalized, continuous, and comprehensive care. It is of great importance for alleviating negative emotions and improving patients' quality of life.

## Methods

### General information

A retrospective study was conducted on 100 patients who have undergone bariatric surgery at the Affiliated Hospital of North Sichuan Medical College from January 1, 2021, to December 31, 2023. Patients were divided into two groups based on the type of nursing method. Fifty patients receiving conventional care (control group) and 50 patients receiving integrated case management with psychological interventions (experimental group). The inclusion criteria comprised: ① Laparoscopic sleeve gastrectomy performed; ② Surgery conducted by the same senior chief physician; ③ Aged 16–50 years; ④ Complete and authentic data. Exclusion criteria included: ① Comorbidities that may influence the study results; ② History of gastrointestinal surgery or sleeve gastrectomy; ③ Abnormal coagulation or hematopoietic function; ④ Presence of mental or psychological disorders; ⑤ History of alcohol abuse or drug addiction. The study was approved by the local ethics committee (Ethics Committee of Affiliated Hospital of North Sichuan Medical College) (No. 2025ER77-1).

### Research protocol

Preoperatively, high-risk patients are identified through anxiety and depression scales (such as SAS, SDS), and cognitive behavioral therapy (CBT) is used to correct irrational cognition about surgery for psychological assessment and intervention. After surgery, customized psychological support content is pushed through WeChat mini-programs (such as one cognitive reconstruction course per month), and telephone follow-up is conducted every 3 months to adjust the intervention strategy as a long-term follow-up intervention after surgery. Standard perioperative care protocols were implemented for the control group during hospitalization, with follow-up for both groups extending to 12 months post-surgery. The specific implementation of the case management combined with psychological intervention model for the control group is as follows: ① A multidisciplinary case management and psychological intervention team is established, including a chief physician for medical support, a case manager for assessing and communicating with bariatric surgery patients, two bariatric surgeons for surgical planning and medical execution, a psychological counselor for targeted interventions, and a bariatric nurse to implement the care plan, monitor the patient's condition, and evaluate the outcomes. ② Prior to surgery, a comprehensive assessment of the patient's condition is conducted, with active communication to fully understand their psychological state. Targeted psychological counseling is provided for patients with negative emotions. Detailed information regarding the surgery and precautions is explained, and multimedia tools and peer support are used to boost patient confidence, reduce preoperative anxiety and concerns, and establish a positive doctor/nurse-patient relationship. ③ Postoperatively, the team collaborates to create a personalized dietary plan for the patient, assist in resolving emerging issues, provide health education, and document the patient's postoperative condition. ④ At discharge, the patient's relevant indicators are assessed and recorded.

### Observation index

Document the patient's preoperative general clinical data, including gender, age, height, weight, BMI, history of hypertension, diabetes, hyperlipidemia, obstructive sleep apnea (OSA), smoking, and alcohol use. Postoperative information includes the time of first gas passage, first eating, first ambulation, length of hospitalization, and postoperative complications. The case manager needs to measure the patient's BMI at 1, 3, 6, and 12 months post-discharge and collect imaging data to monitor the patient's diet, exercise, and other post-discharge behaviors. Anxiety, depression, and other psychological conditions of the enrolled patients were assessed at the first outpatient visit, the day before surgery, the day after surgery, at discharge, and during follow-up. Based on the scale results, the control group received conventional psychological counseling, while the experimental group received personalized, targeted psychological interventions. The SF-36 Health Survey was used to assess the quality of life of both groups at the first outpatient visit, at discharge, and 1 year post-surgery. The assessment primarily evaluated four aspects: physical functioning, bodily pain, emotional functioning, and social functioning. Each item is scored out of 100, with higher scores reflecting better quality of life for the patient. Statistical analysis was performed on the medical satisfaction, psychological conditions, quality of life, and other data from both groups at different time periods.

### Statistical analysis

Data analysis was performed using SPSS 22.0. Continuous data were expressed as mean ± standard deviation (*x* ± *s*) and analyzed using the *t*-test; categorical data were presented as frequency and percentage and analyzed using the *χ*^2^ test. A *P*-value of <0.05 was considered statistically significant.

## Results

### General information

No statistically significant differences were found between the two groups regarding gender, age, height, weight, BMI, history of hypertension, history of diabetes, history of hyperlipidemia, history of OSA, smoking history, and alcohol history (*P* > 0.05), as shown in Table 1.

**Table 1:** Comparison of general data between the two groups.

Variables	Experimental group	Control group	Statistical measurement	*P*
Gender			0	0.815
Male	13	12		
Female	37	38		
Age (years)	34.420 ± 9.283	34.560 ± 9.481	*t* = −0.075	0.901
Height (cm)	164.660 ± 5.109	164.600 ± 5.111	*t* = 0.059	0.840
Weight (kg)	111.124 ± 17.089	111.240 ± 17.373	*t* = −0.034	0.945
BMI (kg/m²)	40.882 ± 5.109	40.968 ± 5.134	*t* = −0.084	0.984
Hypertension			*x*² = 0.219	0.815
Yes	11	13		
No	39	37		
Diabetes			*x*² = 0.160	0.842
Yes	24	26		
No	26	25		
Hyperlipidemia			*x*² = 0.040	1.000
Yes	24	25		
No	26	25		
OSA			*x*² = 0.042	1.000
Yes	31	30		
No	19	20		
Smoking			*x*² = 0.437	0.660
Yes	16	13		
No	34	37		
Alcohol use			*x*² = 0.174	0.835
Yes	17	19		
No	33	31		

### Comparison of postoperative recovery status

There were no statistically significant differences between the two groups in terms of the time to first flatus, time to first feeding, time to first ambulation, length of hospital stay, and postoperative complications (*P* > 0.05), as shown in Table 2.

**Table 2:** Comparison of postoperative recovery outcomes between the two groups.

Variables	Experimental group	Control group	Statistical measurement	*P*
First flatus time (days)	1.800 ± 0.535	1.740 ± 0.443	*t* = 0.611	0.576
First feeding time (days)	3.660 ± 0.479	3.740 ± 0.443	*t* = −0.867	0.087
First ambulation time (days)	1.660 ± 0.479	1.760 ± 0.431	*t* = −1.098	0.031
Hospital stay duration (days)	4.160 ± 0.370	4.260 ± 0.443	*t* = −1.224	0.015
Postoperative complications			*t* = 0.343	0.557
Yes	1	2		
No	49	48		

### Comparison of BMI at 1, 3, 6, and 12 months after surgery

In the comparison of BMI at 1, 3, 6, and 12 months post-discharge, the experimental group had a greater decrease in BMI at 12 months post-surgery than the control group, with a statistically significant difference (*P* < 0.05), as shown in Table 3.

**Table 3:** Comparison of BMI at 1, 3, 6, and 12 months after surgery.

Postoperative period	Experimental group	Control group	Statistical measurement	*P*
1 month	37.656 ± 4.214	37.836 ± 4.347	*t* = −0.210	0.836
3 months	37.041 ± 2.999	36.464 ± 2.878	*t* = 5.936	0.085
6 months	36.419 ± 3.092	36.572 ± 2.535	*t* = −1.626	0.203
12 months	24.340 ± 3.650	27.593 ± 2.535	*t* = −26.446	<0.001

### Comparison of postoperative psychological evaluations

In the comparison of psychological assessments between the two groups, the anxiety and depression scores of the experimental group were higher than those of the control group at the first outpatient visit and at discharge, with statistically significant differences (*P* < 0.05), as shown in Tables 4, 5.

**Table 4:** Anxiety scoring table for both groups.

Variables	Experimental group	Control group	Statistical measurement	*P*
First outpatient visit	56.360 ± 4.530	58.740 ± 5.473	*t* = −2.369	0.021
One day before surgery	60.500 ± 5.179	60.900 ± 5.842	*t* = −0.362	0.544
One day after surgery	56.620 ± 3.948	56.800 ± 3.823	*t* = −0.232	0.737
Discharge	51.420 ± 3.866	52.100 ± 5.567	*t* = −0.709	0.026

**Table 5:** Depression score table for the two groups.

Variables	Experimental group	Control group	Statistical measurement	*P*
First outpatient visit	51.540 ± 3.358	54.560 ± 4.682	*t* = −3.706	0.023
One day before surgery	55.980 ± 3.390	56.280 ± 3.207	*t* = −0.455	0.735
One day after surgery	58.640 ± 3.250	59.040 ± 2.626	*t* = −0.677	0.324
Discharge	49.940 ± 2.653	50.620 ± 1.640	*t* = −1.542	0.019

### Comparison of psychological evaluations

In the quality of life evaluation, the experimental group outperformed the control group in physical function, bodily pain, emotional function, and social function at 6 months post-surgery, with statistically significant differences (*P* < 0.05), as shown in Table 6.

**Table 6:** Comparison of the quality of life evaluation tables for the two groups.

Variables	Experimental group	Control group	Statistical measurement	*P*
Physical function				
First outpatient visit	68.040 ± 5.143	68.260 ± 5.360	*t* = −0.209	0.619
Discharge	67.220 ± 5.456	66.900 ± 4.683	*t* = 0.315	0.664
Six months after surgery	82.600 ± 3.226	82.060 ± 2.289	*t* = 0.965	0.007
Bodily pain				
First outpatient visit	80.960 ± 2.267	81.220 ± 1.930	*t* = −0.617	0.354
Discharge	76.160 ± 3.093	75.700 ± 2.644	*t* = 0.799	0.250
Six months after surgery	81.640 ± 2.777	80.620 ± 4.526	*t* = 0.820	0.019
Emotional function				
First outpatient visit	72.260 ± 2.117	71.860 ± 1.604	*t* = 1.065	0.317
Discharge	67.400 ± 3.136	67.060 ± 3.254	*t* = 0.532	0.669
Six months after surgery	78.420 ± 2.900	78.200 ± 1.050	*t* = 0.504	0.007
Social function				
First outpatient visit	66.460 ± 2.589	66.260 ± 2.656	*t* = 0.381	0.830
Discharge	67.360 ± 2.812	67.280 ± 2.821	*t* = 0.142	0.987
Six months after surgery	76.560 ± 2.101	75.660 ± 1.303	*t* = 0.986	0.003

## Discussion

Bariatric surgery has become a relatively mature discipline after years of development, with its clinical applications and scientific understanding continuously refined through decades of empirical development (7). However, due to obesity and the lack of relevant professional knowledge, patients still express negative emotions such as concern and skepticism about the effectiveness and prognosis of bariatric surgery (8). Systematic review evidence substantiates that structured psychological interventions significantly influence postoperative recovery trajectories, while case management emerges as a coordinated care paradigm demonstrating improved long-term outcomes in chronic disease management (9). The new model based on case management combined with psychological intervention is rare in domestic studies. It is hypothesized that applying this combined model to bariatric surgery patients will have a positive effect on postoperative outcomes and quality of life, making it of great practical value.

In our study, it was found that case managers developed personalized psychological intervention plans based on the patients’ physical and psychological conditions, helping them reduce negative emotions such as anxiety and depression, thus putting their bodies in a better stress state, which facilitates wound healing and physical recovery (10). In this study, no differences were found between the two groups in terms of postoperative recovery and complications (*P* > 0.05), possibly because the surgical techniques at our hospital are well-established, the nursing team is experienced, and bariatric surgery itself is minimally invasive with quick recovery, leading to no statistical differences in postoperative indicators between the two groups. A more evident difference is the more optimized weight control in the experimental group (*P* < 0.05), which may be attributed to preoperative psychological interventions that help patients change negative eating behaviors and attitudes after surgery, such as emotional eating, improving adherence to dietary plans, and achieving better weight control outcomes (11). Additionally, case managers enhance patients' self-efficacy through psychological intervention, allowing patients to see the positive impact of their efforts and changes during the weight loss process, such as weight reduction and improvement in physical function, thus boosting their confidence in controlling weight and managing health. This leads to greater active participation in the recovery process, and the experimental group showed greater weight loss at 12 months post-surgery (*P* < 0.001), reaching the clinically defined “effective weight loss” standard (BMI decreased > 5%) (12). In terms of psychological status, the experimental group had better psychological evaluations preoperatively and at discharge than the control group, the anxiety score of the experimental group was 0.58 points lower than that of the control group (*P* = 0.026), and the depression score was 0.68 points lower (*P* = 0.019). Although the numerical difference was small, it reached the “clinically significant improvement threshold” of the anxiety and depression scale (score reduction > 10%), and was consistent with the patient's self-reported “improvement in emotional management ability”. Bariatric surgery patients often experience psychological stress due to changes in body image and lifestyle adjustments. Psychological interventions, including cognitive-behavioral therapy, help patients properly view the surgery and body changes, manage negative emotions, enhance psychological adaptability, and reduce the incidence of psychological issues such as anxiety and depression (13). Regarding quality of life, the experimental group showed significantly better evaluations than the control group. At 6 months after surgery, the physical function score of the experimental group was 0.54 points higher than that of the control group (*P* = 0.007), and the social function score was 0.9 points higher (*P* = 0.003), indicating that the daily activity ability of the experimental group patients was actually improved. Case management combined with psychological intervention can improve both physical and mental health, leading to improvements in patients' physical, psychological, and social functions. David L. A.'s meta-analysis reviewed 44 articles (representing 36 studies), and the results indicated that social-psychological interventions affect eating behaviors (such as binge eating and emotional eating) and psychological functions (such as quality of life) (14). Thus, case management combined with psychological intervention helps patients engage more actively in social activities post-surgery, improve interpersonal relationships, enjoy life, and increase overall quality of life.

However, it should be acknowledged that our study still has certain limitations. First, the retrospective non-randomized design may lead to implicit confounding (such as higher baseline compliance of patients in the experimental group), and subsequent studies may use propensity score matching or prospective randomization; in addition, psychological assessment and quality of life rely on subjective reports of patients, and in the future they can be combined with objective indicators (such as body fat percentage, sports bracelet data); finally, our samples came from a single center, and the baseline characteristics of the patients were good (such as no serious complications), so caution should be exercised when generalizing our research results to other centers. In conclusion, preoperative case management combined with psychological intervention, through multi-dimensional physiological, psychological, and social interventions, significantly improves postoperative outcomes in bariatric surgery patients. In the future, it is necessary to optimize the intervention plan and promote the integration of precision medicine and psychological support.

## Data Availability

The datasets presented in this article are available upon reasonable request. Requests to access the datasets should be directed to the corresponding author, norshafarina@msu.edu.my.
